# Re-orienting health systems through a commissioning approach: finding solutions for improved consumer engagement

**DOI:** 10.1186/s12961-019-0471-9

**Published:** 2019-07-22

**Authors:** Karen Gardner, Helen Dickinson, Katie Moon

**Affiliations:** Public Service Research Group, University of New South Wales, PO Box 7196, Canberra, BC 2610 Australia

**Keywords:** Consumer engagement, commissioning, stewardship, strategic procurement, primary healthcare, consultation, participation, negotiation

## Abstract

As many developed health systems grapple with the reorientation of their systems to those that are commissioning led, consumer engagement has emerged as an important theme. Despite many governments asserting the importance of consumer engagement in commissioning, an evidence base is yet to be developed to support this approach. This paper identifies the challenges and gaps in the literature relating to consumer engagement and commissioning, before setting out five potential solutions to these challenges. Ultimately, consumer engagement needs clarity of purpose and any approach should be tailored to context. Effective client involvement needs time and investment. To embark on such a process without this effort can be counterproductive.

## Introduction

An ongoing challenge for many developed health systems is how to re-orient from a hospital-centric system to one that more effectively integrates primary and acute care [1]. Across the globe, policy-makers are attempting to design reform processes that will drive greater efficiency and effectiveness in health service delivery, improve coordination of different parts of the health system, counter professional dominance and make more transparent resource allocation decisions. Health systems are increasingly supporting these shifts through a focus on strategic planning and funding or stewardship functions – also known as commissioning approaches [2]. A dominant debate within the commissioning literature is how might we most effectively engage consumers in these processes [3, 4].

In this paper, we draw on a recent review of the international literature on consumer engagement in commissioning [5], setting out some of the trends and considerations for policy-makers and practitioners in developing high quality consumer engagement in commissioning processes and practices. For the purposes of the review, we defined ‘consumer’ broadly to include those who use health services as well as consumer organisations that have a representational or advocacy function. We found that the literature was limited in empirical evidence, despite a strong rhetorical commitment by many governments to both commissioning and consumer engagement. In the next section, we set out the background to commissioning and consumer engagement. We then provide an overview of the five main challenges and gaps we identified within the existing literature. In thinking constructively about how commissioners respond to these gaps, we move the conversation beyond discussing problems by presenting five broad solutions to consumer engagement in commissioning.

## Background: commissioning and consumer engagement

**Fig. 1:**
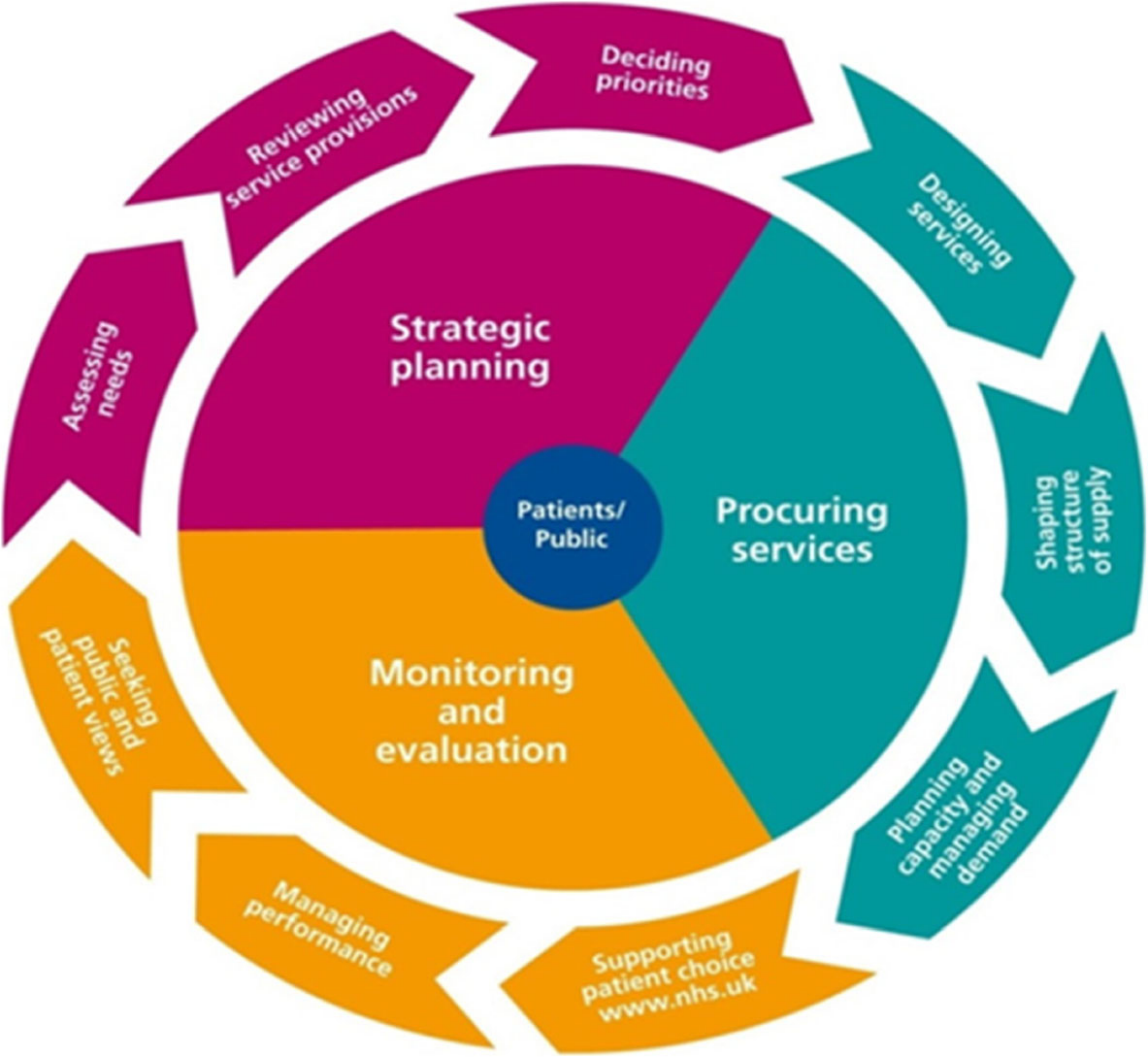
Commissioning cycle reproduced from Tindana et al. [12]

Commissioning has been taken up widely across a number of countries, including Australia, New Zealand, United Kingdom, Germany, the Netherlands and Finland. Although debate concerning the precise definition of commissioning continues, at its simplest commissioning is seen as “*the process of planning, agreeing and monitoring services … Commissioning is not one action but many, ranging from the health-needs assessment for a population, through the clinically based design of patient pathways, to service specification and contract negotiation or procurement, with continuous quality assessment*” [6]. Commissioning is a series of activities concerned with improving processes of health service design and delivery and also holding providers to account for outcomes (at both individual and population level). Commissioning is a complex set of activities and is typically illustrated as a cycle of activities (Fig. 1).

While there is no single model of commissioning and no evidence that any single model achieves better outcomes than others [7], adopting a commissioning approach requires a raft of technical skills, techniques and methods. It also involves a fundamental shift in thinking – from a focus on programmes and their outputs to a more consumer-centred approach that engages with user needs and outcomes at every step [8]. Although engaging consumers is agreed to be an important activity of commissioning processes, inadequate data and advice exists on how effective engagement is achieved. As Coleman and Checkland [9] note, being in favour of more consumer involvement is like being against sin; at a rhetorical level it is hard to find disagreement. The literature, however, indicates a universal lack of clarity in purpose, variation in approaches and processes, and little research into the presumed effects of consumer engagement [10]. Moreover, debate continues over how it is even possible to measure consumer engagement and outcomes over time [11]. Despite these challenges, numerous governments have already committed to consumer engagement to achieve high quality care and outcomes [12].

### Dominant challenges and gaps in the literature

As demonstrated by Dickinson et al. [5], there is a paucity of evidence of commissioning outcomes in both the peer reviewed and grey literatures, even when using a broad definition of ‘evidence’. This paucity extends to both the quantity and quality of this data. Within the existing account, a noticeable gap relates to the evidence of the effectiveness of commissioning in improving client outcomes [5]. Yet, within the limited evidence available, we did identify an important distinction between two broad categories of perceived benefits of consumer engagement in commissioning, namely (1) benefits for consumers participating in commissioning processes and (2) improvements in services. Although a few studies suggested an effect of the benefits for consumers, even fewer demonstrate improvements to services. Where indicated, these few studies include improvements to service environments (e.g. decor, food) and access to services [13, 14].

Although attempts have been made to engage consumers in all parts of the commissioning process, the majority of evidence derives from the parts of the commissioning cycle that deal with making changes to relatively minor aspects of service provision, rather than engaging in strategic planning at a population level. This focus has left some commentators wondering whether consumers have been engaged with commissioning processes at all if we see commissioning as largely concerned with strategic planning and procurement [15]. Scherer and Sexton [16] expand on this point, explaining that experience to date concentrates on provision and development of existing services, because the focus is often on what currently takes place rather than informing or shaping future provision. The same activities could be undertaken within a system that does not operate a commissioning approach. Therefore, questions remain as to whether improvements have been driven by commissioning or by improvements in processes of service provision.

Our synthesis revealed five ongoing challenges to consumer engagement in commissioning processes that are commonly discussed in the literature [5]:

1. Lack of clarity about what consumer engagement means within the context of commissioning [17, 18].
2. Lack of empirical evidence to demonstrate the most effective ways to engage consumers and describe the anticipated outcomes of that engagement [19].
3. Lack of evidence of the skills and competencies that professionals and consumers require to engage with commissioning activities [20, 21].
4. Limited number of established methods to engage representation of different groups and sectors in commissioning processes [22, 23].
5. A range of challenges associated with conducting meaningful and effective engagement [24, 25].

### Broad solutions for consumer engagement in commissioning

Most of the literature included in our review comprised some form of discussion of the abovementioned challenges. What was significantly less common, and typically absent all together, were any lessons, recommendations or solutions to these challenges, limiting opportunities for commissioners to be effective in their engagement activities. In response, we go beyond this literature to describe five broad solutions for consumer engagement in commissioning. These solutions do not individually correspond to the challenges set out above, but instead cut across them in an effort to help equip commissioners to become more effective in their approaches to consumer engagement.

#### Commissioners should be clear about who they are seeking to engage and for what purpose

Commissioning approaches typically seek to engage different stakeholders at various parts of the process, often for quite different purposes. It is important, therefore, that commissioners are clear about who they are seeking to engage and for what purpose (Table 1). This approach ensures that any initiatives are appropriately planned, but also that they do not set up a false sense of what is to be achieved. As Watt et al. explain, “*Getting local people on-board only to let them down, once again, acts as a further step towards disempowerment. Local people … can only feel less powerful through devoting their time, energy and enthusiasm into a project which is later abandoned … by the ultimately more powerful party*” ([26], p. 126).

**Table 1:** Types of consumer engagement

Type of engagement	Purpose	Examples
Communication	To provide consumers with information	Reports, plans, presentations, meetings
Consultation	To obtain consumer and potential consumer ideas, suggestions, complaints and feedback, as well as published consumer research	Paper-based and web-based questionnaires, workshops, focus groups
Negotiation	To reach mutually agreed decisions	Proactive engagement and discussions with consumer forums, membership and/or leaders of projects, steering groups, monitoring groups
Participation	To work together to accomplish commissioning decisions Consumers represented on and actively engaged in all stages of the commissioning cycle	Designing and implementing research Active involvement and responsibility as members of board, ‘mystery shopper’, etc.

Adapted from [27], p. 347

#### Representation warrants consideration

Representation is critical in creating effective commissioning processes. As O’Shea et al. describe, “*representation warrants greater attention, because when it comes to making decisions there will always be a few who decide on behalf of others*” ([18], p. 485). They argue that insufficient attention is sometimes paid to those who are engaged or on whether these individuals are broadly representative of the groups that are sought; this point is particularly true with respect to those who are most seldom heard. A range of different means can be used to achieve representativeness, depending on who or what needs representation. In some contexts, a narrow characteristic (e.g. gender, age and ethnicity) may be appropriate, but it is important that minority groups do not become marginalised in these processes [18]. Where characteristic sharing is not the chosen course it may be because this approach is not an important factor, or that an individual is being asked to act on behalf of another person or group for decision-making purposes. Here, the responsiveness and accountability of the representative to those they represent are important considerations. The crucial takeaway point is that representation is an essential issue and we need to think through who or what we are asking people to represent if individuals and groups are to be appropriately engaged.

#### Be aware of inequalities

Consumers and professionals often experience disparities in terms of the control they have over the design and delivery of services. Millar et al. found that “*if inequalities are not addressed as part of involvement itself this can perpetuate injustice, reinforcing a lack of respect, lack of power and lack of resources. It can also isolate service users, instead of providing opportunities for their mutual support and empowerment*” ([28], p. 215). This point draws attention to the potentially negative implications of consumer engagement within a literature where the aspirations are typically positive. Some professionals, in particular, may find consumer engagement a challenge to their expert beliefs and thus care needs to be taken to ensure that it is possible to engage consumers in a way that avoids undue hostility from professionals, particularly where this approach challenges conventional service delivery practice. It should perhaps go without saying, but corporate commitment, in the form of leadership, resources and strategies, is essential [22]. Additionally, as Schehrer and Sexton [16] remind us, it is important that commissioners do not exceed their authority or fail to carry through on commitments, because doing so can lead to distrust in the process.

#### Embed consumer engagement in the entire organisational change agenda

If consumer engagement is to bring about change, how it will be embedded within the entire organisational change agenda needs to be carefully thought through. Effective consumer engagement that goes beyond a ‘tokenistic’ approach and genuinely seeks to engage consumers is time-consuming and can be difficult to achieve. As an example of this, Albortz et al. [23] studied English Primary Care Trusts and the extent of their consumer engagement processes. Primary Care Trusts were legally mandated to “*communicate and consult with local people*”, but an assessment of these processes found that, “[a]*fter 18 months of operation, more than two-thirds of PCG/Ts* [Primary Care Groups/Trusts] *(69%) had written plans for public involvement, and four out of five (81%) had a public involvement committee or working group. However, only around a fifth of these committees or working groups (21%) had a designated budget and most budgets were £5000 or less*” ([23], p. 22). A number of organisations found it difficult to generate meaningful engagement activities in an expedient way, and many engagement processes remained at a relatively low level. Ultimately, “[b]*est practice in user involvement implies a whole systems approach to ensure that participation/involvement becomes a part of daily life rather than a one-off activity for the whole organisation — from senior management to frontline staff*” ([16], p. 18). Such a task involves a significant change of culture, in addition to thinking about the processes of the organisation in a different way.

#### Engaging consumers in commissioning processes takes time and resources

Evans et al. found, quite simply, that time is one of the greatest resources for effective consumer engagement in commissioning – it is a “*long-term process and often is more challenging and takes more time than professionals initially anticipate*” ([29], p. 513). Time is needed for genuine engagement for training and relationship building. Where engagement is poorly planned and executed, it risks setting up “*a vicious cycle of cynicism about future involvement; by contrast, well planned and well conducted involvement can lead to a virtuous cycle of valuing and therefore investing in involvement*” ([29], p. 513). Involving consumers early in the planning stages before commissioning groups are formally established is offered as a positive approach, although the reality is that it can be difficult to achieve in many cases. Commissioning processes, for example, could include guidelines around remunerating consumers for their engagement. Engagement can be difficult and claim much in terms of emotional resources. Many projects start out with good intentions about engagement but plans are not always realised, particularly because of different and often conflicting priorities. Albortz et al. [23] argue that common methods in use (e.g. newsletters, public meetings, focus groups, questionnaires) are largely ineffective and that more experiments are required to develop and adopt innovative approaches. What this research suggests is that consumer engagement activities must be entered into carefully and with the appropriate resources. As Sanders et al. comment, “[e]*ngagement must be genuine — bad engagement is more damaging than no engagement*” ([11], p. 17).

## Conclusions

Ultimately, consumer engagement needs clarity of purpose and any approach should be tailored accordingly. Effective client involvement needs time and investment. To embark on such a process without this effort can be counterproductive. A lack of evidence relating to commissioning and consumer engagement is challenging in terms of informing the development of these approaches. Few concrete examples are provided in the literature from which commissioners or providers can draw. In an attempt to provide greater guidance to those working in this space we have generated the principles that are set out in the paper to start to address this gap. This lack of evidence does, however, afford an exciting opportunity to build our knowledge around commissioning and consumer engagement processes. It continues to be important to invest in rigorous measurement that includes looking at what activities are effective in improving client outcomes. This work will require access to data and capacity-building to support the use of data in continuous improvement efforts as well as in the measurement and management of progress towards achieving outcomes. Development of data governance arrangements and system architecture may be needed to facilitate feedback processes that support continued engagement of consumers and improvement aims. Relationship building is critical to managing and supporting consumer engagement at different points in the commissioning cycle. New approaches to collaboration and the development of capacities to support these processes will be essential. It should be remembered that commissioning is a young field, as are attempts at widespread consumer engagement. Over the next few years it is likely that the field will develop and grow in terms of the evidence base, provided, in part, that we achieve consistency in terms of how engagement and commissioning processes are described and measured.

## Data Availability

Not applicable
